# Household modifications after the indoor residual spraying (IRS) campaign in Mozambique reduce the actual spray coverage and efficacy

**DOI:** 10.1371/journal.pgph.0000227

**Published:** 2022-04-20

**Authors:** Mercy Opiyo, Ellie Sherrard-Smith, Arlindo Malheia, Arsenio Nhacolo, Charfudin Sacoor, Ariel Nhacolo, Mara Máquina, Luis Jamu, Nelson Cuamba, Quique Bassat, Francisco Saúte, Krijn Paaijmans

**Affiliations:** 1 Barcelona Institute for Global Health (ISGlobal), Hospital Clínic - Universitat de Barcelona, Barcelona, Spain; 2 Centro de Investigação em Saúde de Manhiça (CISM), Maputo, Mozambique; 3 MRC Centre for Global Infectious Disease Analysis, Imperial College London, London, United Kingdom; 4 National Malaria Control Programme of Mozambique (NMCP), Ministry of Health, Maputo, Mozambique; 5 PMI VectorLink Project, Abt Associates Inc., Maputo, Mozambique; 6 ICREA, Barcelona, Spain; 7 Pediatrics Department, Hospital Sant Joan de Déu, Universitat de Barcelona, Esplugues, Barcelona, Spain; 8 Consorcio de Investigación Biomédica en Red de Epidemiología y Salud Pública (CIBERESP), Madrid, Spain; 9 Center for Evolution and Medicine, School of Life Sciences, Arizona State University, Tempe, Arizona, United States of America; 10 The Biodesign Center for Immunotherapy, Vaccines, and Virotherapy, Arizona State University, Tempe, Arizona, United States of America; 11 Simon A. Levin Mathematical, Computational and Modeling Sciences Center, Arizona State University, Tempe, Arizona, United States of America; London School of Hygiene & Tropical Medicine, UNITED KINGDOM

## Abstract

Indoor residual spraying of insecticides (IRS) is a key malaria vector control strategy. Whilst human attitude towards IRS is monitored before or shortly after implementation, human activities leading to the modification of insecticide-treated walls post-IRS are not. This could inadvertently reduce the protective effects of IRS. We monitored the extent of modifications to the sprayed indoor wall surfaces by household owners for six months post-IRS campaigns in two districts targeted for malaria elimination in southern Mozambique. In parallel, we assessed building of any additional rooms onto compounds, and mosquito net use. We quantified the contribution of wall modifications, added rooms, prolonged spray campaigns, and product residual efficacies on actual IRS coverage and relative mosquito bite reduction, using a mechanistic approach. Household owners continually modified insecticide-treated walls and added rooms onto compounds. Household surveys in southern Mozambique showed frequent modification of indoor walls (0–17.2% of households modified rooms monthly) and/or added rooms (0–16.2% of households added rooms monthly). Actual IRS coverage reduced from an assumed 97% to just 39% in Matutuine, but only from 96% to 91% in Boane, translating to 43% and 5.8% estimated increases in relative daily mosquito bites per person. Integrating post-IRS knowledge, attitude, and practice (KAP) surveys into programmatic evaluations to capture these modification and construction trends can help improve IRS program efficiency and product assessment.

## Introduction

According to the World Health Organization (WHO), Mozambique is among the five countries accounting for more than half of all malaria cases globally [1, 2]. Malaria is largely endemic throughout the country and even though significant reductions in malaria burden have been observed in the past two decades [1, 3] it is clear that progress has stalled in recent years [2, 4]. Malaria vector control in the country relies mainly on two complementary cornerstone interventions that are implemented on a large scale: indoor residual spraying (IRS) and long-lasting insecticide-treated nets (LLINs) [5, 6]. Alongside these tools, increased surveillance, and improved access to health care are also promoted by the National Malaria Control Programme (NMCP). Whilst LLINs are distributed widely throughout the country [7], IRS is normally implemented in specific geographic settings to mitigate insecticide resistance and/or aid the rapid disruption of malaria transmission [4, 6, 8, 9].

For IRS, given its wider array of chemical classes compared to those approved for LLINs, several large studies have assessed, or are investigating, when and where IRS in combination with LLINs will result in additional reductions in malaria morbidity and mortality [9–11]. However, the efficacy of both interventions can be significantly reduced by factors such as sub-optimal coverage or usage of the interventions, widespread insecticide resistance, and malaria mosquitoes resting/biting outdoors [6, 12–14]. As the widespread resistance to pyrethroids—the most common class used in LLINs—continues to be a challenge, and economic costs to deliver IRS fall, IRS may take a more central role in malaria control in several settings across the African continent [9, 15].

To assess the impact of IRS after implementation, the residual effect of the IRS product, as well as the coverage achieved at the end of the spray campaign are reported and used in models [15–17]. This assumes that reported coverage is achieved within a short time and does not change post-IRS implementation although spray efficacy is observed and modelled to decrease due to a loss in residual efficacy over time [15, 18].

The threshold coverage at which IRS provides community protection (i.e. also protecting people living in unsprayed compounds/structures) is believed to be ~85% of all households in a given area [16, 19, 20]. This coverage is almost unanimously reported as the proportion of households protected by the end of the spray campaign. It is therefore assumed that a) sprayed surfaces are not modified and b) no structures are added onto the households once the IRS campaign has been completed. But any modification to sprayed household walls (such as painting, replastering, scrubbing, washing or the addition of large wall decorations) as well as the construction of new (and thus unsprayed) rooms would likely reduce or negate effective sustained pressure on the vector populations by the IRS active ingredient. While this human-driven indicator is crucial for the effectiveness of the IRS in the period post-application, it is less studied and its effects less understood [19, 21–23]. Two earlier studies from India and South Africa demonstrated that initial IRS coverage may decrease rapidly after IRS is implemented as a result of human-related activities performed within the personal household spaces such as painting, washing, and brushing of the insecticide-treated walls [24, 25]. These human-driven activities may greatly affect the effectiveness of an IRS product by removing the insecticide or by reducing its bioavailability. As a result this may reduce the number of households effectively covered with IRS in a community, which will more than likely impact the community-wide epidemiological efficacy of this intervention [21, 24, 25].

In addition, campaigns can take months to complete as it is unfeasible to prepare, visit and spray all households within a month in a particular district, and community engagement is needed to iteratively increase the overall proportion of households covered [19]. This also means that at the completion of the IRS campaign, those households sprayed first may have a lower concentration of active ingredient than those households sprayed towards the end of the IRS campaign, due to the reductions in the killing efficacy of insecticides over time [15], with starker differences if campaigns last longer. This is due to the fact that IRS products have a certain residual efficacy, which is estimated to be 2–6 months for pyrethroids, DDT and carbamates, and 6–9 months for the next-generation products [25, 26]. As the active ingredient wanes, so too do their protective effects for the individuals of the recipient household and the community more broadly. These potential drivers of variation in temporal IRS efficacy between households are not currently evaluated and are not generally considered in assessing the campaign’s overall public health impact.

To guide routine monitoring and to improve IRS usage, and hence its impact, the aim of this study was to quantify and assess: i) the impact of human-related activities conducted within personal household spaces post-IRS application, and; ii) the change in assumed protective efficacy that may result from the prolonged deployment of IRS across a community given variable concentration of insecticide in households over time. This study builds upon the limited evidence explaining IRS protection variability and opens an avenue to investigate a number of important questions on indoor-based vector intervention use. We report empirical data on the modification of insecticide-treated walls and the addition of rooms to households post-indoor residual spraying (IRS) activities in two malaria elimination districts in southern Mozambique. Households were monitored for a period of six months after the implementation of IRS, and households’ members answered questions about the extent and type of modification (washing, painting, brushing, replastering) to the walls in the various types of household rooms, the number, and type of newly constructed rooms, the use of LLINs, the use of wall decorations and their opinion about vector control tools. Subsequently, we quantified the contribution of wall modifications, additional rooms, the prolonged spray campaigns, and the products’ residual efficacies on the actual IRS coverage and corresponding potential change in mosquito bites received per person over time, using a mechanistic framework informed by both our empirical data as well as known estimates for various mosquito and intervention parameters.

## Methods

### Ethics statement

The study was reviewed and approved by Comité Institucional de Bioetica para Saude do CISM (CIBS-CISM/038/2018). Participation in the study was voluntary and the study interviewees were free to leave the study at any point in time. Household heads provided written informed consent.

### Study area

**Fig 1:**
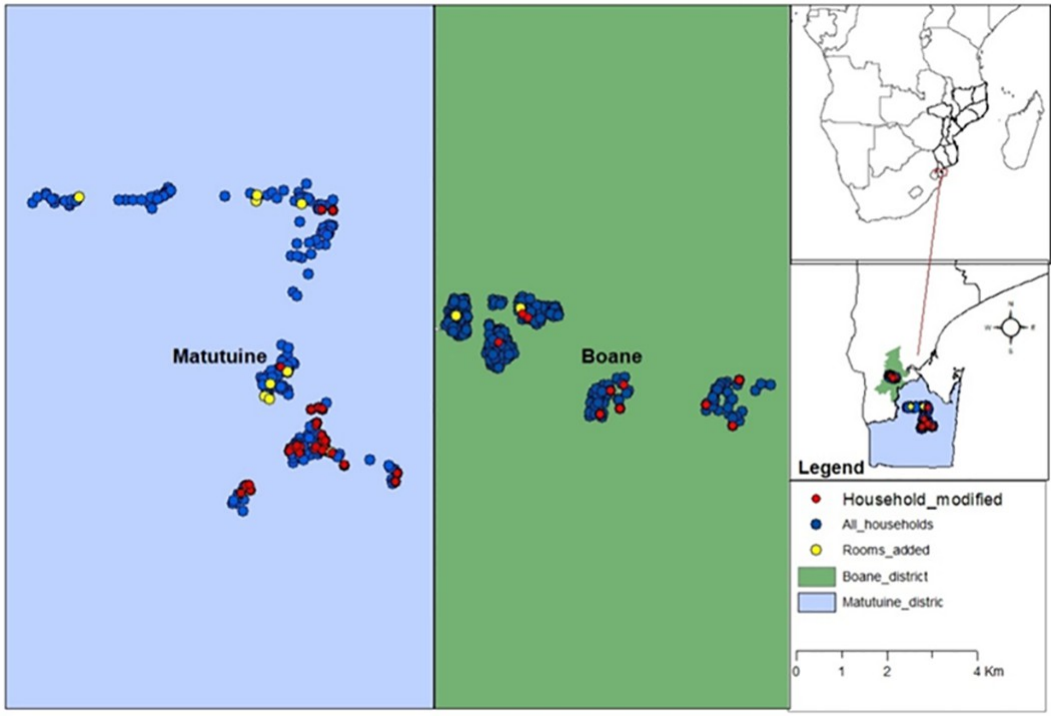
Map of Matutuine and Boane districts in southern Mozambique showing households with walls modified (red dots) and rooms added (yellow dots) to the households. The base layer was obtained from: https://data.humdata.org/dataset/mozambique-administrative-levels-0-3.

The study was conducted from October 2018 to July 2019 in 8 sub-villages in Matutuine and Boane districts in Maputo Province, southern Mozambique (Fig 1). In Matutuine district, the study villages were Vila de Bela Vista, Missevene, Salamanga, Tinonganine, and Madjuva. In Boane, the study was conducted in Vila de Boane, Manguiza, and Gueguegue. Both the communities predominantly practice mixed agricultural small-scale farming of crops such as cassava and maize. The study villages in Matutuine and Boane are mostly within rural and peri-urban areas. Briefly, the region has a year-round malaria transmission with seasonal peaks during and after rainy seasons (December-April) [27]. The study districts were purposely selected based on the history of continuous IRS implementation in these regions since 1999 as well as accessibility [28]. IRS operation campaigns in southern Mozambique are routinely conducted by the National Malaria Control Programme of Mozambique (NMCP) in collaboration with the Goodbye Malaria Initiative (GBM). The two districts were chosen to allow us to assess any contrasting human behavior affecting wall surface management given the two different IRS products principally used in the country since 2018. Matutuine was sprayed with the organophosphate active known as Actellic and Boane with a neonicotinoid active, called SumiShield. Currently, the districts are sprayed with different insecticides each year as part of the NMCP’s insecticide resistance management strategy, which follows the WHO recommendations [29]. The key malaria vector species responsible for malaria transmission in both districts has not been monitored recently. However, historic data suggest that *Anopheles gambiae* and *An*. *funestus* species complexes are most prevalent in Matutuine (*personal communication*, *NMCP Programme*, *Mozambique*) while *An*. *merus*, and *An*. *arabiensis* are key species in Boane [30]. The study was conducted for six months and was launched in conjunction with the NMCP annual IRS campaign in the southern part of the country. The IRS campaigns started in August 2018 and ran until February 2019 the following year. Household selections for this study started in October and continued to December due to logistical challenges. Households were recruited as IRS was rolled out, and only recently sprayed (5 days old) households were recruited in the study for follow-up.

### Study design

Five hundred and ninety households were purposely selected randomly across 8 sub-villages in Matutuine and Boane districts (Fig 1). Only adjacent villages were selected to facilitate study implementation. A semi-structured questionnaire was administered to the head of each household; an adult above 18 years of age. This questionnaire allowed us to assess the frequency of wall modification, type of wall modification, the reason for wall modifications, added rooms or structures since the IRS campaign, perception of IRS, vector control method preferences, and mosquito net usage. The same households were followed every month for six months after enrolment. Questions were asked in the local language, Portuguese or Shangana. Data collectors were employed from the local study areas and they were well conversed with the culture of their localities to ensure understanding in the respective communities. An additional senior supervisor was also included to ensure smooth field activity operation and timeliness and quality of interviews. Data were collected by an electronic questionnaire using Open Data Kit software (ODK; https://opendatakit.org). Data analysis was done using statistical software R version 4.0.2 (R Core Team, Vienna, Austria). Further simulation analysis was conducted to assess the impact of post-spray wall modifications and prolonged IRS campaigns on the actual operational IRS coverage over time (at the household level), the residual efficacy of the IRS products as well as the effect on the mosquito biting rate on people. Only relevant data to assess the impact of housing modification and added rooms/structures post-IRS implementation were analysed and presented as explained below.

#### Information on the IRS campaigns

IRS was implemented by the NMCP in collaboration with GBM. In Matutuine, where the organophosphate IRS product was used, the NMCP aimed to cover 21,992 houses, but 15,453 houses were visited and of those 14,780 sprayed. The campaign started in August and was completed in January. In Boane, where the neonicotinoid IRS product was implemented, the NMCP aimed to cover 55,947 houses, but 58,635 houses were visited of which 56,611 were sprayed. The campaign started in August and was completed in February. A household is considered eligible for IRS if there is/are closed structure(s) with a ceiling, that are habitable, and have suitable walls/surfaces for IRS products. The latter includes surfaces made of mud, cement, reeds, straw, wood, and/or bamboo, and include painted surface.

Household coverage can be defined in two ways; i) Coverage of planned households, which is the numbers of households sprayed divided by those that were anticipated, or ii) Coverage of visited households, which is the number of sprayed households divided by those visited. As information on a household-level is not available (only at the house-level, see above), and the number of anticipated houses can be severely over- or underestimated, as it is based on older census data, we report the coverage of those houses that were actually visited, which is 96% for Matutuine and 97% for Boane, and assume this is equivalent to household coverage in our model.

#### Impact of wall modification and prolonged spray campaigns on actual operational IRS coverage

Traditionally, estimates from spray campaigns tend to assume that the reported overall household level coverage achieved by the campaign once completed contributes the same level of protection to all sprayed households from a single time point (campaign completion date) [15, 31]. But while some households may have a single bedroom, others may have many, and if the household is reported to have been covered by the spray campaign, it is not always clear whether all eligible spaces are sprayed. Further, these campaigns may take months to deliver in reality depending on factors such as geographical settings, and acceptance rate in the targeted region. However, the protective efficacy of IRS products wanes from the day of application onward. So, households are afforded different levels of protection depending on when the house/structure was sprayed with knock-on impacts for community protection (as insecticides kill mosquitoes, which reduces the community risk of being bitten in addition to providing protection to the occupants of the recipient compound).

We use a mechanistic framework adapted from Griffin et al. [22] to investigate how the observed changes in IRS effective coverage in Matutuine and Boane districts over time affect the effectiveness of each IRS candidate. For the analysis and to demonstrate the impact of a spraying schedule that stretches over multiple months, we use the weekly data from the spray campaign teams on the percentage of households sprayed, assuming that if a household is covered it receives full protection (regardless of whether some rooms are missed). We combine these data with the accumulated monthly data in Table 1 on modified households monitored from November onward—assuming that modification covering at least 75% of the wall surfaces negates any protective effect from the IRS—to estimate the actual IRS cover overtime. For example, in November in Matutuine about 92% of the spray campaign was completed (IRS deployment started in the 3^rd^ week of August 2018). In November, 14 households out of 129 sampled had been modified (Table 1). Therefore, at this particular time the actual IRS cover—the proportion of households protected by IRS in November—was about 10% less than the campaign reported (Table 1).

**Table 1:** Monthly household observations from Matutuine and Boane districts. The number of households visited for 6 months post-IRS to observe modifications to sprayed wall surfaces, or added rooms to the households, and the consequential adjusted IRS cover for communities receiving spray in October, November, or December 2018. Corresponding reported LLIN use is shown for each cohort. The total IRS coverage reported on the completion of the campaign was 96% in Matutuine and 97% in Boane, though this was accumulated across 4 months (mid-August 2018 to December 2018).

Month of households selection (month of IRS application)	Month of interviews	Month post-IRS	No. of households successfully assessed		No. of households with new modifications since last visit (% of M1 households assessed)		No. of households adding rooms since the last visit		Adjusted cumulative IRS coverage accounting for modifications and added rooms (%)		Reported LLIN use (%)	
			Matutuine	Boane	Matutuine	Boane	Matutuine	Boane	Matutuine	Boane	Matutuine	Boane
Oct2018	Nov 2018	M1	129	113	14 (10.9%)	4 (3.5%)	0	0	89.1%	96.5%	27.9%	57.5%
	Dec 2018	M2	117	89	7 (5.4%)	0 (0.0%)	10	1	77.7%	95.6%	32.6%	64.6%
	Jan 2019	M3	117	88	20 (15.5%)	2 (1.8%)	19	1	55.7%	93.0%	43.4%	68.1%
	Feb 2019	M4	116	86	20 (15.5%)	0 (0.0%)	15	1	39.3%	92.2%	55.0%	62.8%
	March 2019	M5	115	85	12 (9.3%)	0 (0.0%)	12	0	30.3%	92.2%	61.2%	70.8%
	April 2019	M6	114	83	13 (10.1%)	0 (0.0%)	12	1	21.8%	91.5%	51.9%	65.5%
Nov2018	Dec2018	M1	88	153	12 (13.6%)	4 (2.6%)	0	0	86.4%	97.4%	42.1%	74.5%
	Jan2019	M2	86	144	10 (11.4%)	2 (1.3%)	7	2	69.4%	94.8%	43.2%	71.9%
	Feb2019	M3	85	141	10 (11.4%)	1 (0.6%)	9	4	53.8%	91.8%	44.3%	71.2%
	March 2019	M4	85	138	7 (8.0%)	0 (0.0%)	11	0	42.6%	91.8%	56.8%	82.4%
	April 2019	M5	84	137	6 (6.8%)	1 (0.6%)	11	2	34.1%	90.1%	61.4%	81.1%
	May 2019	M6	81	134	4 (4.6%)	0 (0.0%)	9	1	28.9%	89.5%	48.9%	78.4%
Dec2018	Jan 2019	M1	27	76	4 (14.8%)	3 (4.0%)	0	0	85.2%	96.1%	33.3%	67.1%
	Feb 2019	M2	25	75	3 (11.1%)	1 (1.3%)	1	1	71.4%	93.5%	48.2%	79.0%
	March 2019	M3	25	75	1 (3.7%)	2 (2.6%)	1	0	65.5%	90.9%	40.7%	86.8%
	April 2019	M4	25	75	1 (3.7%)	0 (0.0%)	1	1	60.0%	89.7%	66.7%	81.6%
	May 2019	M5	25	74	1 (3.7%)	0 (0.0%)	0	0	56.7%	89.7%	55.6%	84.2%
	June 2019	M6	14	9	0 (0.0%)	0 (0.0%)	1	0	54.8%	89.7%	29.6%	10.5%

LLIN use was monitored throughout the study in the sentinel districts. A considerably higher proportion of households used LLINs in Boane (mean 64.4%; range: 10.5%– 81.3%, Table 1) relative to Matutuine (mean 43.6%; range: 27.9%–60.0%, Table 1) and in both districts LLIN use varied monthly (Table 1). The most recent LLIN mass distribution campaign took place between 2016 and 2017 [32], which means the nets would be approx. 1.5 years old during this study. This means that the protection afforded by nets will have waned slightly relative to the performance of a new net. This can be captured following Churcher et al. for pyrethroid-LLINs in the absence of resistance [33].

#### Impact of wall modification post-IRS campaigns on actual operational IRS coverage

Household modification here refers to any changes made on the insecticide-treated walls such as painting, plastering, washing walls, or other modifications that would render the IRS product ineffective or reduce the effectiveness of IRS. The actual IRS operational coverage (demographic coverage or initial coverage) here refers to the coverage achieved in the field in a targeted region (in our case in Matutuine or Boane districts) during IRS implementation or campaigns, often reported by the NMCP or the implementing entity. That is, the proportion of households sprayed given the prior understanding of all potential households targeted within the district. The total accumulated IRS coverage (IRS started in August 2018 and ended in February 2019) during the 2018/2019 campaign was reported to be 96% household coverage in Matutuine and 97% household coverage in Boane (see above). We refer to IRS usage as the adherence of owners of the households to retain (manage the insecticide-treated walls without modification of any kind); and the protection offered by intact IRS-treated surfaces over time as the IRS effective or actual coverage. With modification to households over time, the IRS usage and cover reduces (Table 1).

#### Predicted impact of wall modification and prolonged IRS campaigns on the potential for malaria transmission

**Fig 2:**
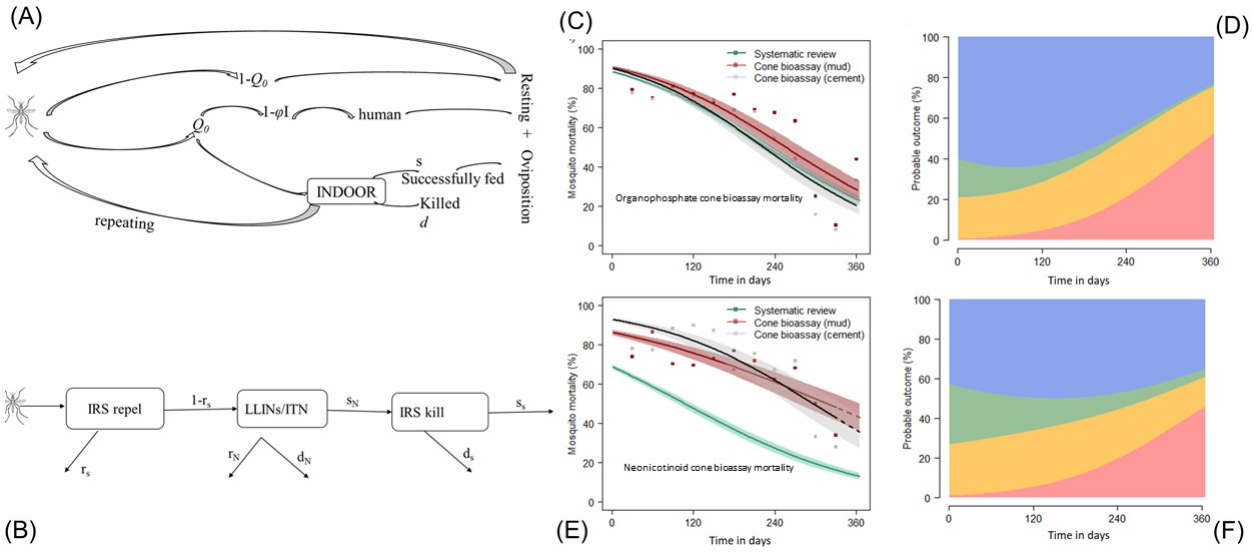
*Anopheles* mosquito feeding attempt in Magude (sprayed with an organophosphate active) and Manhica (Palmeira) (sprayed with a neonicotinoid active) districts. (**A** & **B**) Mosquito feeding cycle and how it is impacted by IRS and mosquito nets [20, 22]. Panel (**A**) shows a mosquito may feed with some probability on an animal (1 –*Q*_*0*_) or human (*Q*_*0*_). If seeking a human, the mosquito may feed indoors (probability *φ*_*I*_) or outdoors (1 − *φ*_*I*_). Mosquito nets (LLINs), or IRS alters the probability of a successful blood meal (*s*) indoors and the probability of mosquito mortality whilst attempting to feed (*d*). Mosquitoes that are neither fed or killed can repeat (*r*) the pathway on the next feeding attempt. Subscript *N* indicates net induced impacts and *S*, mortality induced by IRS. Panel (**B**) shows the assumed pathway of a mosquito indoors and the protective action of nets and IRS. (**C**) Cone bioassay tests were assessed over time since spraying using fully susceptible *An*. *arabiensis* KGB colony mosquitoes in Magude district (an organophosphate active). (**E**) Cone bioassay tests were done using fully susceptible *Anopheles arabiensis* KGB colony mosquitoes in Manhica district (Palmeira) (a neonicotinoid active). On average, the proportion of mud to cement households in Matutuine as assessed by our survey was (34%) and (54%) and other materials (12%) in Boane districts, households were mud (97%) and cement (3%) respectively. For **C** and **E**, the proportion of mosquitoes killed during the bioassay is shown for mud (red) and cement (black) walls. A logistic binomial regression (line shows median; polygon shows 95% credible intervals) is fitted using Bayesian analysis to describe the time-dependent waning mortality effect of each chemistry on either mud (red) or cement (grey) walls. The corresponding median mortality curve for each active ingredient from a systematic review is shown in green [18]. Each line indicates the prediction given empirical data (solid) confirming the fit or onward where we are less certain (dashed). Panels (**D**), and (**F**) show the average predicted the probable outcome of an *Anopheles* mosquito feeding attempt; mosquito mortality (blue), deterred before entering (green), repelled without feeding (orange), or successfully feeding (red) for Matutuine (organophosphate active), and Boane (neonicotinoid active) districts, respectively.

Indoor interventions are assumed to act on mosquito vectors by i) deterring blood-seeking females from entering treated houses; ii) repelling mosquitoes before feeding once indoors; iii) or killing mosquitoes resting indoors before or after feeding (Fig 2A) [16, 20, 22]. The probability of mosquitoes successfully blood-feeding (feeding and surviving) is reduced by indoor interventions. Together, IRS and LLINs protect the whole community by reducing mosquito densities so, at high effective coverage, more people are protected with a consequent reduction in the risk of infectious mosquito bites. Whilst mosquito net campaigns tend to be delivered *en mass* (within a week) using assembly points or community health care centres for community members to access new mosquito nets, spray campaigns are inevitably slower to deliver as it takes time to recruit households, for households to accept the intervention and empty their houses, and to physically spray all eligible rooms of each household. Therefore, IRS effective coverage gradually increases over the course of subsequent months. Modifying indoor wall surfaces after a spray campaign will reduce the effective coverage of the IRS, the number of people protected, and could have a substantial impact on malaria transmission. Further, the surface of the household walls may have an impact on the rate of decay of the active ingredient in the sprays [34, 35]. Results from a systematic review [18] for either organophosphate-based or neonicotinoid-based IRS candidate data were modified using local cone bioassay data, weighted for the proportion of houses that are constructed with either mud or cement, to estimate the probable outcomes in Matutuine and Boane districts of southern Mozambique (organophosphate IRS (Fig 2C and 2D), and neonicotinoid IRS (Fig 2E and 2F)). We explore how the observed changes in IRS effective coverage in Matutuine and Boane districts over time affect the effectiveness of each IRS candidate given the respective LLIN use in each region. We estimate the changes in the mosquito biting rates on people that result from prolonged campaign delivery and wall modification practices across 6-months in the two districts relative to a scenario where we assume the IRS is delivered overnight and is retained (actual or absolute coverage) as reported at the start of the IRS campaigns.

#### Mechanistic analysis of IRS effective coverage.

To conceptualize the impact of post-spray wall modification, prolonged spray campaigns, addition of rooms onto households and IRS efficacy on IRS effectiveness, we adapt a probabilistic vector model approach determined in Le Menach [20] and Griffin [22]. This model outlines how indoor interventions are affecting the probability that a person receives a mosquito bite per unit time which has ramifications for the infectious mosquito bites received per person per year (the entomological inoculation rate, EIR) and malaria transmission. The probability that a blood-seeking mosquito successfully feeds will depend on the species-specific bionomics and behaviors of the mosquito (e.g. the proportion of bites taken on humans, the proportion of bites received indoors or in bed) and the vector interventions that protect the human population. In our case, in the absence of locally available mosquito bionomics data, we use a uncertainty analysis to explore potential impacts from these estimated parameters (Table 2). In the presence of LLINs in the household (an LLIN can either kill or repel the mosquito), repelled mosquitoes are assumed to attempt to find alternative blood meal sources (a process referred to as repeating (r)). Following Churcher et al. [33], we use the parameters determining the efficacy of mosquitoes when entirely susceptible to pyrethroid insecticide but after LLINs are 1.5 years old reflecting the timing of the IRS monitoring study (Oct 2018 onward) relative to the most recent LLIN mass campaign (2016–2017) [32]. We assume that all livestock are kept outdoors and therefore all mosquitoes that enter the house are attempting to bite humans.

**Table 2:** Mechanistic model parameters for the estimation of mosquito bites per day. Parameter estimates represent a generic “An. funestus-like” mosquito.

Parameter	Symbol	Estimate
Anthropophagy	*Q*_*0*_	0.92 (0.78–0.98) [53]
Proportion of bites taken on humans indoors [15]	*Φ*_*I*_	0.90 (0.68–0.99) [54]
Proportion of bites taken on humans in bed [15]	*Φ*_*B*_	0.85 (0.60–0.95)
Baseline time spent looking for a blood meal	*δ*_10_	0.69 days
Time spent resting between feeds	*δ*_2_	2.31 days
Probability of surviving the host-seeking process without interventions	*p*_*10*_	0.913
Probability of surviving the resting period	*p*_*2*_	0.737

The estimates in this table follow the derivations in Griffin et al. [22] unless otherwise noted.

#### Time-dependent effect of IRS active ingredient

The probabilities that a mosquito will be killed (*l*_*s*_) or successfully blood-fed (*k*_*s*_) or neither (*j*_*s*_) have been assessed previously for organophosphate and neonicotinoid IRS candidates using the systematic review of experimental hut data [18] and are conditional on mosquitoes not being deterred before entering a sprayed hut (Fig 2). We repeat the analysis here for completion. A flexible time-dependent logistic function is fitted to experimental hut data for each product separately:

$$l_{S}=\frac{1}{1+\text{exp}\left(-\left(l_{S\vartheta }+l_{S\gamma }\;\times t\right)\right)}$$ (1)

$$N_{dead}\sim binomial\left(l_{s},\;N_{total1}\right)$$ (2)

$$k_{S}=\frac{k_{0}}{1+\text{exp}\left(-\left(k_{S\vartheta }+k_{S\gamma }\;\times t\right)\right)}$$ (3)

$$N_{successfully\_fed}\sim binomial\left(k_{s},\;N_{total1}\right)$$ (4)

$$m_{S}=\frac{1}{1+\text{exp}\left(-\left(m_{S\vartheta }+l_{S\gamma }\;\times t\right)\right)}$$ (5)

$$N_{deterred}\sim binomial\left(m_{s},\;N_{total2}\right)$$ (6)

Parameter *l*_*S*_ denotes the proportion of mosquitoes being killed given initial efficacy (*l*_*Sϑ*_) and impact duration (*l*_*Sγ*_) across *t* days since the spray deployment. The logistic model is fitted using the total number of mosquitoes killed (*N*_*dead*_) in the sprayed huts (*N*_*total1*_). The proportion of mosquitoes successfully feeding (*k*_*S*_) and being deterred away from a sprayed hut (*m*_*S*_) are similarly determined. Deterred mosquitoes (*N*_*deterred*_) are calculated from the difference between mosquitoes in control and sprayed huts. The proportion of mosquitoes that enter and are then repelled without being killed or feeding is then *j*_*s*_ *= 1 –l*_*s*_*−k*_*s*_. Previously the parameters for the transmission model have been fitted and estimate *k*_*0*_ as 0.699 [22]. The *k*_*s*_ fits are scaled to ensure that the probabilities that a mosquito entering a sprayed hut successfully blood-feeds, exits without feeding, or dies, denoted *s*_*S*_, *r*_*S*_ and *d*_*S*_ respectively, are within the 0 to 1 range.

The functions *k*_*s*,_ *l*_*s*_ and *j*_*s*_ are adjusted by the degree of deterrence (*m*_*s*_, a time-varying quantity) as follows,

$$l\prime_{S}=l_{S}\times \left(1-m_{S}\right)$$ (7)

$$k\prime_{S}=k_{S}\times \left(1-m_{S}\right)$$ (8)

$$j\prime_{S}=j_{S}\times \left(1-m_{S}\right)+m_{S}$$ (9)

We then estimate the probability that mosquitoes are biting successfully (*s*_*S*_), repeating (*r*_*s*_), or being killed (*d*_*s*_) as:

$$s_{s}=\frac{k\prime_{S}}{k_{0}}$$ (10)

$$r_{s}=\left(1-\frac{k\prime_{S}}{k_{0}}\right)\times \left(\frac{j\prime_{S}}{\;l\prime_{S}+j\prime_{S}}\right)$$ (11)

$$d_{s}=\left(1-\frac{k\prime_{S}}{k_{0}}\right)\times \left(\frac{\;l\prime_{S}}{\;l\prime_{S}+j\prime_{S}}\right).$$ (12)

Because these effects are time-dependent, the probability of a mosquito being killed is continually waning from the moment of application onward. We carry through uncertainty from the logistic fits using 50 iterations from the posterior draws 90% credible intervals.

#### Site-specific IRS efficacy given effective IRS coverage

The methodology and the results of cone bioassay data used to assess the impact of housing modification on the IRS will be described elsewhere (Fernández-Montoya *et al*., *in review*). In the absence of data to better inform this approach, we make a simplifying assumption that the cone bioassay mosquito mortality estimates recorded in Matutuine and Boane over 6-months is equivalent to the mortality measured in experimental hut trials so that we can translate the probable outcome for a mosquito feeding attempt from these data using the analysis of a systematic review [18] (Fig 2). The cone bioassay mortality in this study is consistent with the estimated impact from previous systematic reviews using experimental hut mortality to infer the durability of IRS products as described in (Fig 2). Therefore, we adjust the relative probabilities for biting and successfully feeding (*s*_*S*_), repeating (*r*_*s*_), or being killed (*d*_*s*_) estimated in [18], whilst taking into account different killing rates on mud or cement surfaces in Matutuine (Fig 2C) and Boane (Fig 2E). Once again, we track the uncertainty from the posterior draws of the original fits. The time-dependent efficacy from LLINs is estimated similarly following [22] where the subscript *N* denotes the successful biting (*s*_*N*_), repeating (*r*_*N*_), and killing (*d*_*N*_) in the presence of LLINs and assuming the effects have waned given 1.5 years since the mass campaign by the time the IRS monitoring period begins.

#### Estimating mosquito bite rates in the presence of modified wall surface post-IRS implementation

Following Le Menach [20] and Griffin [22], we assume that the probability a mosquito bites an individual-host (*i*) during a single attempt is (*y*_*i*_); a mosquito bites a host and survives the feeding attempt is (*w*_*i*_), and the probability that it is repelled without feeding is (*z*_*i*_). These probabilities exclude natural vector mortality, so that for an individual with no protection, *y*_*i*_ = *w*_*i*_ = 1, and *z*_*i*_ = 0. With IRS or LLINs, these estimates are altered which consequently reduces the relative mosquito bites received per person per year (Table 3).

**Table 3:** Vector control probability matrix courtesy of Griffin et al. [22].

	IRS only	LLINs only	IRS plus LLIN
Probability of successful feeding, *w*_*i*_	1 − *φ*_*I*_ + *φ*_*I*_(1 − *r*_*S*_)*s*_*S*_	1 − *φ*_*B*_ + *φ*_*B*_*s*_*N*_	1 − *φ*_*I*_ + *φ*_*B*_ (1 − *r*_*S*_) *s*_*N*_*s*_*S*_ + (*φ*_*I*_ − *φ*_*B*_)(1 − *r*_*S*_)*s*_*S*_
Probability of biting, *y*_*i*_	1 − *φ*_*I*_ + *φ*_*I*_ (1 − *r*_*S*_)	1 − *φ*_*B*_ + *φ*_*B*_*s*_*N*_	1 − *φ*_*I*_ + *φ*_*B*_ (1 − *r*_*S*_) *s*_*N*_ + (*φ*_*I*_ − *φ*_*B*_)(1 − *r*_*S*_)
Probability of repellency, *z*_*i*_	*φ*_*I*_ *r*_*S*_	*φ*_*B*_ *r*_*N*_	*φ*_*B*_ (1 − *r*_*S*_) *r*_*N*_ + *φ*_*I*_*r*_*S*_

In Table 3, *Φ*_*I*_ and *Φ*_*B*_ are the proportion of bites taken on humans indoors or in bed [22]. The average probability of a mosquito successfully feeding during a single attempt is:

$$W=\left(1-Q_{0}\right)+Q_{0}\Sigma_{i}w_{imb}$$ (13)

Here, *w*_*i*_ is the probability of success on an attempt to feed on the *i* person in a population. $Q_{0}^{v}$ is the proportion of bites taken on humans by mosquito species *v* in the absence of any intervention. The probability of the mosquito being repelled is:

$$Z=Q_{0}\Sigma_{i}z_{imb}$$ (14)

The value of *w*_*i*_ and *z*_*i*_ will differ depending on each starting week of a spray campaign *m* for representative households. We adjust these estimates (W and Z) to account for the effects of prolonged IRS deployment over multiple months by weighting the estimate by the observed proportion of households assumed to be newly covered by the spray campaign each month. To include the household modification, we simply multiply the monthly estimates of the *w*_*i*_ and *z*_*i*_ by the appropriate IRS effective cover (*b*) observed in the assessment period (November 2018 to July 2019). We demonstrate how the probabilities determined in Table 3 are altered by these adjustments.

We assume that human movement and sleeping patterns are not dependent on age or relative exposure in the absence of more informative data. In this instance, for simplicity, as we intend here to conceptualize the impact of IRS cover, we also ignore mosquito species-specific effects. The feeding rate is defined in the presence of indoor interventions as:

$$f_{R}=\frac{1}{\frac{\delta_{10}}{1-Z}+\delta_{2}}$$ (15)

*δ*_*10*_ represents the average time spent host-seeking in the absence of indoor vector control and *δ*_*2*_ is the time that the mosquito spends resting after feeding. These are assumed to be constant. The mortality rate is affected by interventions such that:

$$\mu =-f_{R}log\left(\frac{Wp_{10}}{1-Zp_{10}}p_{2}\right),$$ (16)

where *p*_*10*_ is the probability of surviving the host-seeking process without interventions and *p*_*2*_ is the probability of surviving the resting period. We can then estimate the proportion of successful bites on people as:

$$Q=1-\frac{\left(1-Q_{0}\right)}{W}$$ (17)

The rate at which a person in the population is bitten by mosquitoes (the number of bites received per person per day) is then calculated as:

$$\lambda_{i}=\frac{Qf_{R}y_{i}}{\sum_{i}w_{i}}$$ (18)

All estimated parameter ranges used in the framework are provided in Table 2. As we do not have any information about the absolute number of mosquitoes, nor the number or proportion of cases of malaria infection in people in either study district, we instead explore how the relative number of mosquito bites received per person per day is altered by the prolonged deployment of IRS and the household modifications after spraying in Matutuine (sprayed with an organophosphate active) and Boane (sprayed with neonicotinoid active) across the 6-month survey to demonstrate lost protection to the community with housing modification.

## Results

### Summary of wall modification, and the addition of rooms to the households

Here, we define a household as a compound (henceforth we refer to it as a household) where a family lives, and which consists of one or more buildings/structures such as main houses (with indoor bedrooms, living rooms, storage, bathrooms, kitchens, and garages, or other rooms), and/or outdoor buildings/structures such as toilets, kitchens, or animal shelters. In our study, ‘wall modification’ is defined as any activity conducted on the walls by household owners after IRS application such as plastering, painting, washing, or brushing walls post-IRS. We define ‘household modification’ as the wall modification and/or the addition of rooms onto the household—which could be a stand-alone and/or additional room(s) added to a house within a household. In this work, for those households who had any room modified, on average 94% reported that they had all four walls in those rooms altered. All households reporting that rooms had been modified had at least 25% of the wall surfaces modified. We include all reports of modified rooms in the analysis to estimate reduced effective IRS cover across the community.

**Fig 3:**
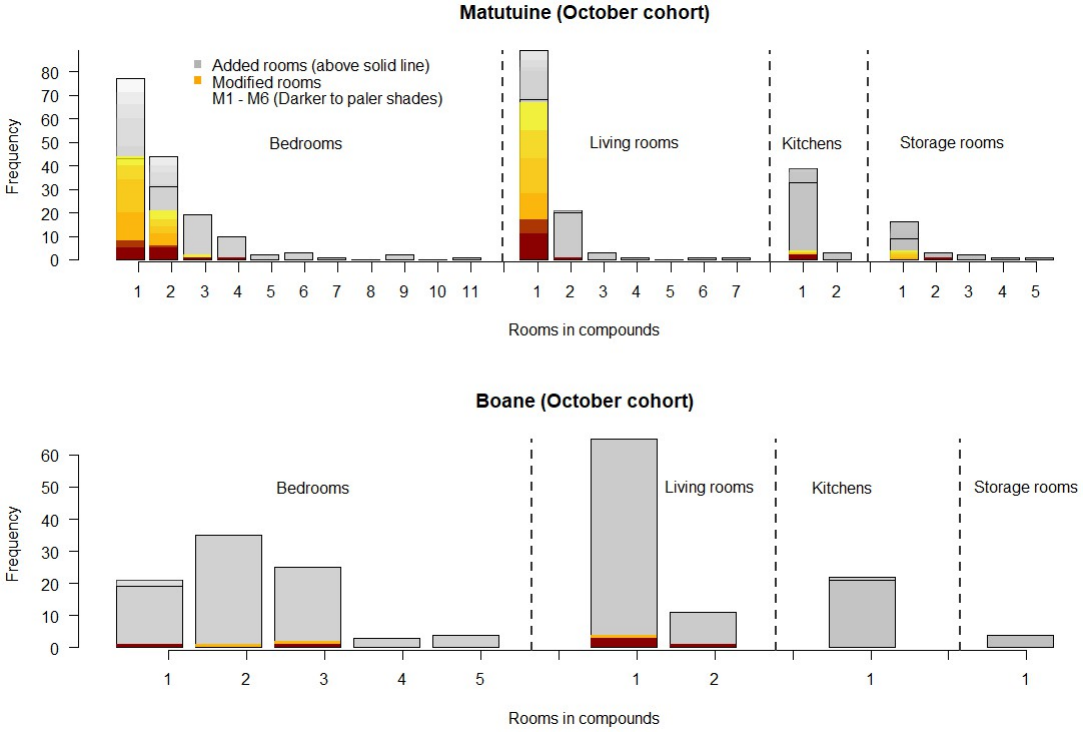
Summary of rooms receiving modification or the addition of rooms to households in the October cohort (see Table 1) for Matutuine (top panel) and Boane (bottom panel). In each panel, the data are divided by room type into bedrooms, living rooms, kitchens, and storage rooms. Shown in grey is the original number of rooms at the start of the study, noted as the first horizontal bar on each block. The upper limit of the block then shows the total rooms after additions. In Matutuine (top panel), the first bar shows that 43 households had one bedroom at the initiation of the study post-IRS. Over time, an additional 44 bedrooms were added to the households with, originally, one bedroom. No bedrooms were added in follow-up month 1, but then 5 were added in month 2, 12 in month 3, and 6 in months 4 to 6 of the survey shown as pale grey bands on the figure. Similarly, for modifications, there were 5 bedrooms modified in the first month after IRS, then 3 more in month 2, then 12, 14, 6, and 4 more modified in months 3 through to 6 shown as shaded orange and red bands on the figure (dark red indicates month 1 and yellow shows month 6). This is repeated for the number and types of rooms reported on each household, no changes were made to households with 5 or more rooms. Repeat figures for cohorts recruited in November and December are shown in S1 and S2 Figs, respectively.

In Matutuine district, we recruited 129 households sprayed in October 2018 (Table 1) with 559 rooms of which 39 rooms in 14 households had been modified by November (Table 1), and no added rooms were reported. By December, a further 7 households from this cohort had modified a total of 11 rooms and 10 households had added a total of 22 rooms to their respective households (Table 1). Across the full follow-up period for the household cohort recruited in October, a total of 175 rooms were modified by April 2019. Of these, 96 were bedrooms and 69 were living rooms. During that same time, a total of 84 rooms were added to households, 48 bedrooms, 16 living rooms, 13 kitchens, and 7 storage rooms. Two further cohorts were recruited, 88 households in November and 27 households in December, each cohort received IRS one month prior to the first interview. In Matutuine, there was an average of 5.5 rooms per household, and for those households that had modification (n = 143), an average of 37.8% (95% confidence intervals: 11.6–76.3%) of the rooms were modified. Fig 3A summarises the room modifications and additional rooms for the first cohort in the Matutuine district. We provide equivalent figures for the 2^nd^ and 3^rd^ cohorts in S1 and S2 Figs.

Households were recruited from Boane in the same way, across months October to December (Table 1). There were far fewer modifications to walls or additional building of rooms in Boane (Table 1 and Fig 3). In total, only 20 out of 342 households made any modification to rooms and only 15 households added rooms. Once again, the most commonly-added rooms were bedrooms (66%) (Fig 3). In Boane, there was an average of 5.7 rooms per household, and for those households that reported modification, an average of 34.9% (95%CI: 8.3–77.7%) of the rooms were modified.

Overall, the household owners mostly used mops (65%) as the tool to wash walls and thus modify the sprayed surface. Principally, water plus detergents (59%), or only water (35%) were applied as the liquids to clean the insecticide-treated surfaces. In Matutuine district, for the ten households who responded to undertaking plastering, 82% (n = 9) did so because of normal maintenance and renovation, the others because of general cleaning (9%, n = 1) and decoration purposes (9%, n = 1). For those 16 households who mentioned washing the walls, 67% (n = 10) did so to remove the product’s smell, 40% (n = 6) to remove insecticide stains left on the walls and only 1 household mentioned that they were asked to wash the walls. When walls were painted (n = 4 households painted walls within a sprayed room), it was for general maintenance and renovation only.

In Boane district, for the 16 households who responded that they had modified walls of sprayed rooms, 83% (n = 5 households) replastered their houses because of general maintenance and renovation, while one household mentioned decoration as the reason for plastering. Six households washed the walls and explained that this was because children were touching the walls (50%, n = 3 households) or to remove the insecticide product’s smell (50%, n = 3). Four households with rooms that were painted did so for decoration purposes.

Regarding wall decorations (hanging fabrics that decorate the room), which oftentimes hang in front of sprayed wall surfaces, 55% of households in Matutuine and 85% in Boane districts had at least one wall partially covered in one room with a fabric (see S3 Fig for examples). We do not consider the effects of such wall hangings in the analysis as most of the room sprayed surfaces were available to mosquitoes, but this represents a further consideration for assessing the efficacy of spraying campaigns.

### Impact of wall modification, the addition of rooms to households, prolonged spray campaigns, and residual activity of IRS product on effective IRS coverage and reduction in mosquito biting rates

**Fig 4:**
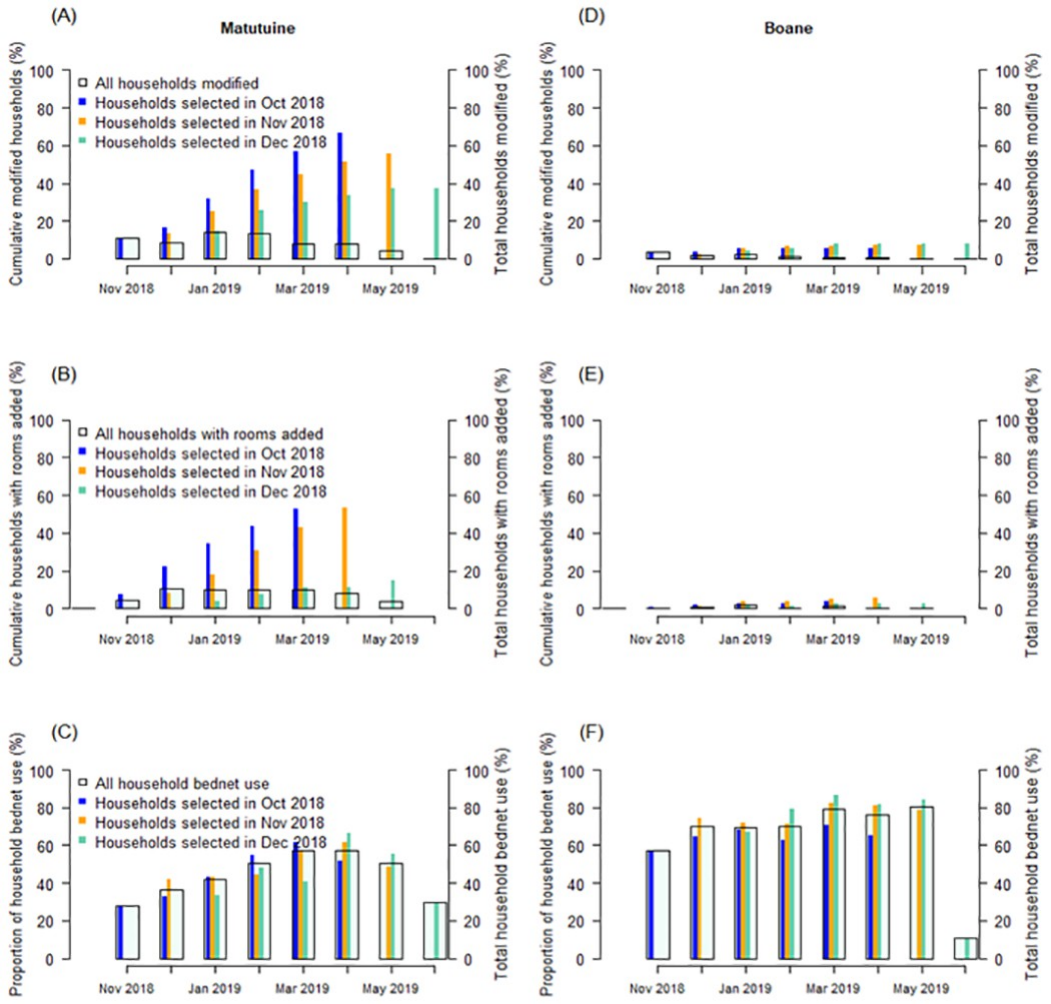
Summary of household modifications post spraying (Number of households assessed monthly are provided in Table 1). In each panel, the colour of the bar indicates the month when the households were first sprayed and recruited to the trial; (October, blue), November (orange), and December (green). The overlaid square with black outline shows the average proportion of households modified (**A** and **D**), rooms added on the compound (**B** and **E**), and mosquito net use (**C** and **F**) for each month during the follow-up. This allows us to determine that e.g. in Matutuine, most household modifications took place in January-February and the peak modification took place from November through February. The accumulation of households with walls modified fully or partly by either painting, washing, or scrubbing over time (wall modification) in (**A**) Matutuine district, and (**D**) Boane district; The accumulation of households with rooms or compartment added on the compound in (**C**) Matutuine district and (**F**) Boane district. Mosquito net use in (**B**) Matutuine district and (**E**) Boane district.

**Fig 5:**
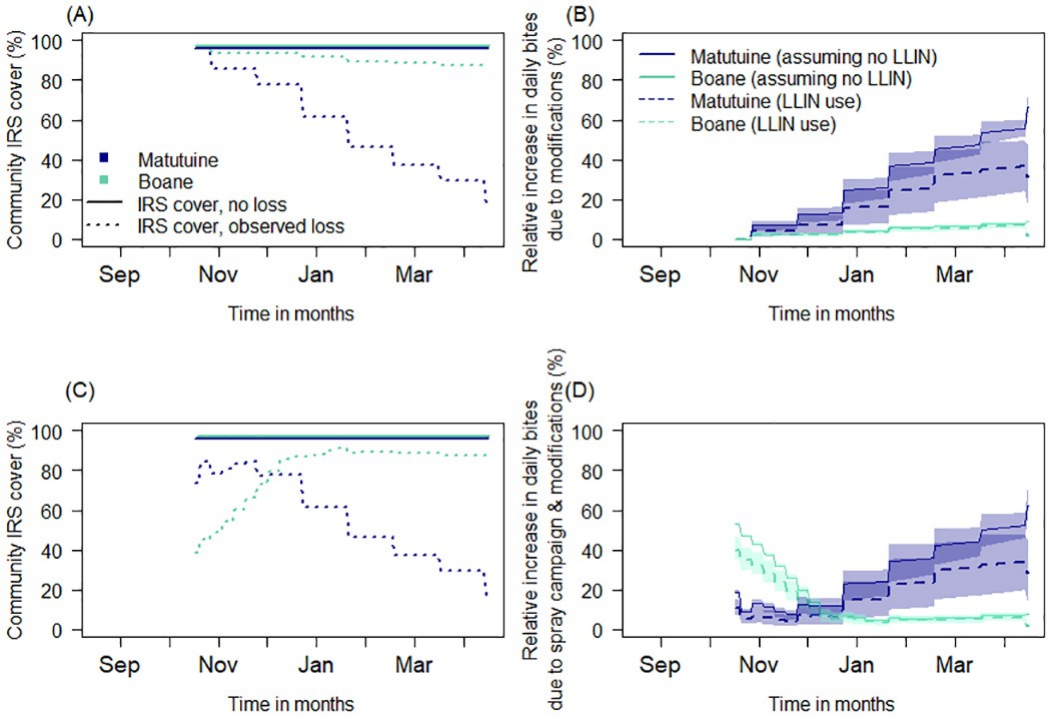
Comparison of factors impacting the IRS effectiveness. Assuming overnight delivery of IRS in October; (**A**). Change in post-IRS spray cover due to room modification or addition (accumulated data in Table 1) in Matutuine (blue) and Boane (green) districts sprayed with an organophosphate active and a neonicotinoid active, respectively; (**B**). The relative increase in daily mosquito biting rates in each district assuming overnight spraying in October in the presence (dashed lines) and absence (solid lines) of mosquito net use. That is, the estimated percentage increase in mosquito bites received per person relative to what we would expect without any household modifications reducing IRS effective cover. In contrast, (**C**). change in post-IRS spray coverage given the spray campaigns are completed over multiple months and household wall modification is ongoing in Matutuine (dotted blue) and Boane (dotted green) districts, compared to assuming IRS cover is as reported at completion and without modifications (solid lines: 96% for Matutuine, blue; or 97.1% for Boane, green); (**D**). Change in mosquito biting rates relative to assuming no loss in protection from prolonged spraying, modifications or added rooms, given the presence or absence of mosquito nets resulting from prolonged spray campaigns. In (B) and (D) the presence of nets mitigates for the potential lost protection from ongoing modifications or use of additional bedrooms (unsprayed). 90% uncertainty intervals are carried through the analysis and reflect IRS product efficacy over time, and the uncertainty analysis for parameters shown in Table 2. Similar estimation is presented assuming prolonged spray campaign in S4 Fig.

To model the impact of the modification activities on IRS, we considered ‘wall modification’ as an umbrella term for any modification (e.g. washed away, plastered, brushed, or painted) to a room or space that is eligible for spraying that would have altered the contact of the mosquito to rest on the sprayed surface. As all modifications affected at least 25% of the sprayed space within that room, and 94% of modifications affected all walls, we included all the data for the analysis (Table 1). Analyses here are based on three key factors: i) the post-spray campaign wall modifications (Fig 4A and 4D) or additional rooms built on the compound (Fig 4C and 4F) that alter the actual coverage of the IRS; ii) the time taken to deliver spray campaigns (17 and 24 weeks in Matutuine and Boane respectively, spray campaign data provided by GoodBye Malaria Initiative (GBM)) for a single district, and; iii) the waning concentration of active ingredients sprayed onto wall surfaces over time. The IRS spray operation of the 2018–2019 period took place across multiple months (we define this as “prolonged spray campaign process”) and, for our analysis, we applied the weekly percentage data provided by the spray team to cumulatively increase IRS cover from mid-August until completion in each district. During this time, the sprayed household walls were progressively modified negating the actual household protection from the spray campaign in some households. Therefore, we can also compare the total IRS cover given these cumulated modifications alongside the spray campaign. The peak household wall modification in Matutuine and Boane districts occurred between November and February (Fig 4A and 4D). If we ignore the prolonged spray campaign process assuming IRS campaigns were complete in October, we observe a reduction in IRS effective coverage due to post-spray household modification (measured from November) of 96% to 16.0% through the monitored period (Fig 5A), with progressively more potential for mosquito bites over time in Matutuine district given the higher rate of wall modifications (ranging from 5% to 17% of the total households recruited per month). In contrast, in Boane district, assuming rapid IRS deployment completed in October, IRS effective cover fell only from 97.1% to 86.9% (Fig 5A). On combining these two processes—a prolonged spray campaign and modification to sprayed households—we find that in Matutuine, IRS effective coverage was greatest in late-November (84.8%) when nearly all (96%) households were assumed to have received spray whilst only 11% (14 of 129 monitored households, Table 1) had been modified (Fig 5C). Boane would likely have had the cumulative highest IRS effective coverage in February onward (90.1%) given the prolonged spray campaign and minimal household modification (Fig 5C).

**Fig 6:**
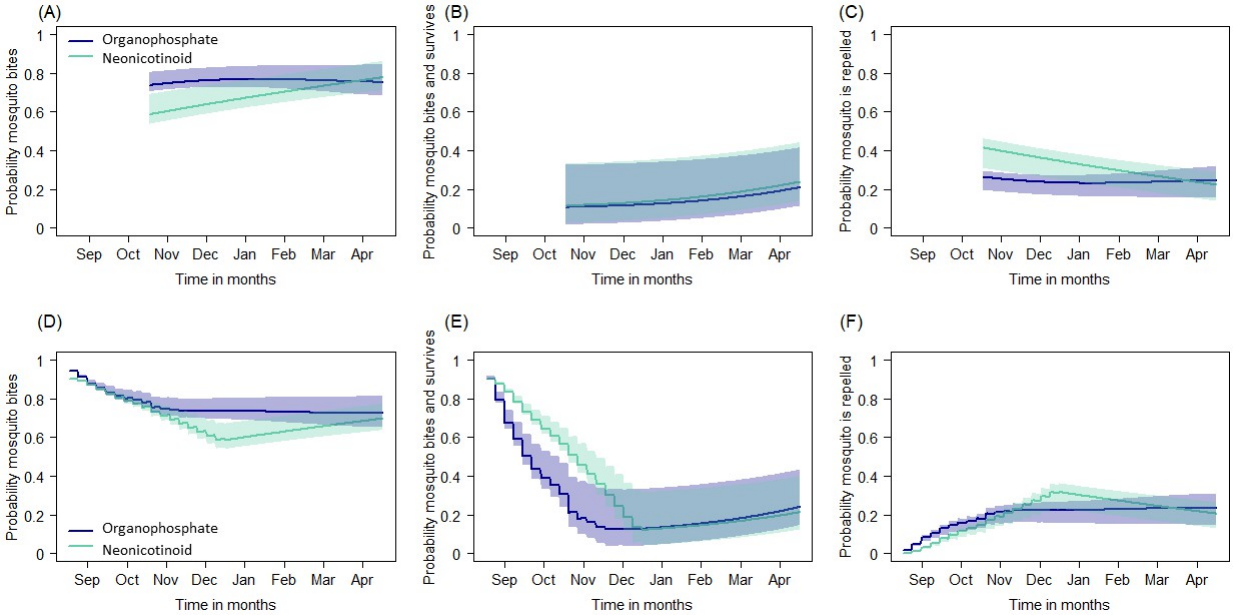
The estimated efficacy of insecticidal sprays wanes with time since delivery. Using the average (and 90% credible intervals) estimated impacts of these sprays [22], we contrast the expected outcomes from mosquito blood-feeding attempts indoors when assuming overnight delivery of the IRS (**A-C**)–so that waning begins in October unanimously—to the impacts expected when accounting for the prolonged delivery of the spray campaign (**D-F**). The probability that a mosquito bites, is higher for the organophosphate IRS candidate that was used in Matutuine (blue) than for the neonicotinoid IRS candidate deployed in Boane (green) in both the overnight (**A**) and prolonged (**D**) scenarios, but the probability that the mosquito bites and survives are fairly equivalent (**B** & **E**) but lower for organophosphate IRS candidate when accounting for the prolonged spray campaigns (**E**). Given the accumulation of protected compounds, the lowest probability of mosquito biting and surviving is achieved in December to January (**B** & **E**). These effects are reflected in the probability that the mosquito is repelled (**C** & **F**).

In the mechanistic model we determine the probability that a mosquito will bite at all, will bite and survive that feeding attempt, and whether it is repelled without biting at all (Fig 6), assuming similar mosquito vector species and vector behaviours in both districts as this information was not collected empirically (see discussion). These probabilities change over time as the concentration of active ingredients in each product wane. Based on a previous analysis [18] the probability of mosquitoes biting at all is distinct for the two active ingredients (organophosphate IRS candidates used in Matutuine; and neonicotinoid IRS candidates used in Boane; Fig 6A), with the neonicotinoid IRS candidate apparently inducing a greater repellence effect (Fig 6C), though there is little difference in the probability of successful bites (Fig 6B). But this previous analysis assumed overnight deployment of the IRS. When we further account for the prolonged distribution of the campaign such that the product deployed is working optimally in the week of deployment and then gradually waning (Fig 6D–6F), we see that the community is best protected, i.e. the probability of receiving bites is lowest in December to January (Fig 6D and 6F), as the insecticide active is most likely to be present at high cover and still potent, so can deter and kill mosquitoes effectively (Fig 6E and 6F), in both districts.

Combining all three processes—a prolonged campaign, the waning impact from IRS products after delivery, and the modification of households (modifying walls or adding rooms) post-spraying—we find interesting effects on the estimated increase in mosquito biting rate on humans observed in each district relative to not having any spraying (Fig 5B and 5D and S4 Fig). Once again, the greatest protection is afforded in December to January, nearly 4 months after the spray campaign was initiated in either district and when the maximum number of households are sprayed and modifications (or additions) continue to be accumulated later in the season.

We quantify the contribution of wall modifications/room additions to reduced protection from mosquito bites and prolonged spray campaigns. With mosquito net use (Table 1), and assuming spraying happens instantaneously in October, we would expect a broadly continuous increase, from maximal protection (0% additional bites) to 41% additional bites in Matutuine, or 0% to 6.7% in Boane, in additional (relative to no wall modification or addition of rooms to households) mosquito bites per person per day by March-April resulting directly from the modifications made to households as documented in Table 1 and Fig 5B and S5 Fig. In Matutuine, the prolonged IRS campaign results in 5% to 38% additional bites per person from November to April, i.e. a fairly similar level of protection during peak mosquito biting season, such that the wall modifications are likely driving the loss in protection (Fig 5D). In contrast, the prolonged spraying in Boane results in an estimated 22% additional relative mosquito bites per day in November, which falls to about 8% in December and 5% in January (Fig 5D). In this case, the prolonged campaign that leaves some community members without IRS protection until February reduces effective IRS cover relative to what would be assumed traditionally (i.e. 97.1% to have been deployed ‘overnight’). Given that the peak transmission is often toward the end of the rainy season in April or May, IRS may not be present to continue killing mosquitoes during these critical months. These two processes—household modification (wall modification and addition of rooms) and duration of the spray campaign—both prove important for assessing and providing effective IRS community protection. Finally, the combination of both higher LLIN use and fewer wall modifications led to a lower overall estimate for the relative increase in mosquito biting rate estimated for people in Boane compared to Matutuine (Fig 5, S4 and S5 Figs) irrespective of the product deployed. This has implications for our assessment of product performance which we will discuss.

## Discussion

Sub-optimal coverage of vector control tools within household spaces threaten to undermine malaria control and elimination efforts. Identifying the factors contributing to sub-optimal tool deployment and understanding this impact on the effectiveness of the vector control is important to enable mitigation strategies to fill protection gaps for recipient communities. The aim of this study was to assess the impact of human activities within personal household spaces (relating to wall surfaces being modified and/or added to the household) on IRS coverage and IRS effectiveness over time. Household wall modification (either by plastering, painting, washing, and brushing) and the building of additional buildings/structures, particularly rooms to sleep in, after IRS campaigns, occurred at different rates in the two districts studied in southern Mozambique. Such household wall modification reduces actual IRS coverage and corresponds to reduced community protection offered by IRS over time. In addition, prolonged IRS campaigns alter the effective coverage and impact of IRS, but this depends on the timing of spray campaigns relative to the seasonality of malaria transmission. Together these factors led to a loss of IRS effective cover in the two districts studied, which is likely to have resulted in increased risk of mosquito bites.

Household wall modification within personal compound spaces post-IRS has also been observed in India in 2005 and South Africa in 2000 [24, 25]. While the studies in India and South Africa only quantified these activities in regions sprayed with DDT and Deltamethrin respectively, our study is the first to assess household wall management post-application and its impact in regions sprayed with Next Generation IRS (NgenIRS) products. We find that the operational (initial) IRS coverage in Matutuine and Boane districts reduced continually across the six months’ post-spray application as a consequence of the changes made to room wall surfaces and the addition of bedrooms by household owners. In Matutuine district, which was sprayed with an organophosphate IRS candidate, the reduction in operational post-IRS coverage was far higher than in Boane where a neonicotinoid IRS candidate was used (Table 1). This comparison of sprayed products is not part of this study, so we do not know if the higher rate of wall modifications is partly driven by product type, although the most common reason given for modifying walls was ‘because of the smell’, and the organophosphate IRS candidate is known to have a strong odor [36]. However, the extent of building of additional rooms for bedrooms, living rooms, and kitchens indicates that Matutuine is a growing community, a reality that would likely be ongoing independent of IRS [37]. From observations and again, not specifically tested in our study, Boane households are closely built together potentially making further constructions less feasible or desirable, and among other socio-economic differences, Boane district is generally more urban than Matutuine. We are also unable to comment on the degree to which the different wall modifications taking place might reduce the efficacy of the sprayed products and future work would be needed to specifically address this. This is an important limitation as we make the simplifying assumption in the analysis that any household modification affecting more than 75% of the wall space results in a total loss of protection from the IRS, which may over-estimate the negative impact of wall modifications for some processes. These anecdotal differences clearly highlight the need for context when comparing the performance of IRS products in community settings. The changes we observe negate the possibility to compare the performance of these two IRS products between these two settings for instance; whichever product was applied in Matutuine—where the community are expanding structures within households regularly, probably irrespectively of IRS campaigns—would likely have reduced effect relative to Boane where structures are more established. Similarly, as we have no data on the density of mosquitoes relative to people in each village we cannot comment on any epidemiological effects. Further work is needed to consider whether the properties of a particular product drive modification behaviours.

The vector control framework used in this study highlights that modified wall surfaces can potentially lead to a substantial increase in the relative risk of mosquito bites. Nevertheless, the intensity of the adjusted impact varies according to the scale of the modification, use of alternative interventions (e.g. mosquito nets), and assumed entomological impact of respective products [18]. In Matutuine district, the relative risk of mosquito bite was substantially higher than in Boane district reflecting comparably fewer modifications made to houses in Boane. Given that the ability of IRS to kill mosquitoes primarily depends on how long the treated surfaces are intact, which in turn is dependent on how long the insecticide remains active on the surfaces, the decline in the efficacy of IRS seen in this study highlights the challenges of community-related activities post-IRS application and the consequential potential gap in protection left by this type of intervention. Moreover, despite the substantial research and modelling exercises assessing the impact of malaria vector control interventions, to our knowledge, none of the existing malaria transmission and vector models have simulated post-IRS coverage changes, prolonged IRS campaigns, or how such changes would affect the number of people protected by the intervention and the overall impact of IRS on malaria transmission [16, 22, 38]. To include these adjustments in a full transmission model such as Griffin et al. [39] is the natural next step for the presented work which could allow us to explore how to achieve optimum protection from IRS intervention whilst accounting for associated logistical challenges.

This study has the following additional limitations. First, it was limited to only two districts in southern Mozambique, and there is a need to extend investigations to other districts and settings across the African continent sprayed with different IRS products particularly as the phenomena we observe are likely specific to each location. As a result, the conclusions cannot be generalized; clearly, the two districts studied were quite different in: i) the time taken to complete the IRS campaign; ii) the proportion of sprayed rooms that were modified over time, and; iii) the proportion of new rooms added to a household over time. These activities are context-dependent but given the growth of housing improvements ongoing across the continent [40], the latter may well contribute to the reduced impact of the IRS (and other) interventions elsewhere. Second, household enrolment occurred over three months and therefore, the study might have missed the peak housing modification periods and underestimated the frequency of such activities in these two communities, although our work suggests households are fairly consistently modified regardless of the month of the year (Fig 4A and 4D). Given the variability in wall modifications in the two districts, it is reasonable to assume there may also be within-district differences that we do not account for. We also present a deterministic framework, fixing certain parameters related to mosquito behaviours (in the absence of more informative data, Table 2) and attempt to mitigate for this limitation by conducting a uncertainty analysis for a realistic range of parameter estimates. This serves the purpose to highlight the altered potential of IRS given delays in delivery of the intervention and household modification but there is further work to be done to look at the epidemiological effects relating to these changes and any uncertainty in these estimates across seasons. It would be important to understand distinct mosquito species behaviors and how they relate to the efficacy of the different IRS products, which we currently do not consider in our mechanistic framework. However, we make explicit assumptions about the behaviour of mosquito species (which are more flexible in reality) and do not allow these to vary seasonally (Table 2). We assume the effects of IRS are equivalent between species (although the encounter rate, that is driven by mosquito behaviour is species-specific within the framework). We also assume that the effects of IRS and mosquito nets are independent, which is yet to be proven (or disproven) using empirical data. We have data aggregated across months for wall modifications and LLIN use, and across weeks for the spray campaign delivery, so the effects, in reality, are likely smoother than depicted in Fig 5, S4 and S5 Figs. We outline the implicit modelling assumptions in the methods. We do not have the absolute entomological or epidemiological data to understand whether the mechanistic framework we apply accurately captures the public health performance of these products under these conditions. There is a clear need to collect entomological, epidemiological, social and intervention data simultaneously, to allow for an adequate impact assessment of vector control interventions on both entomological parameters and pathogen transmission [18]. Future questions include whether different ways of modifying wall surfaces have distinct consequences for IRS efficacy, how generalizable are these wall modifying behaviors in settings with increasing urban growth, and how variable these behaviors are year-on-year? All these caveats aside, we are reporting on real phenomena which are likely to play a role in the performance of these interventions elsewhere in Mozambique and across Africa, particularly given the rate of urbanization in Africa [37].

There are also other challenges for IRS including the consistency of active ingredient concentration sprayed onto walls, the potential for campaigns to miss households during pre-spray census work, community acceptance and misreporting [41, 42]. It is also imperative that future studies (through focus group discussions and in-depth interviews) look into the detailed reasons why communities conduct such activities in regions sprayed with different insecticides, particularly whether the modification is directly related to the product, and whether these could be undertaken before spray campaigns or in the dry season for example.

In this current study, we have focussed on the loss of killing efficacy of IRS insecticides (which is minimal for these long-lasting NGenIRS products in the time frame studied), the loss of coverage as a result of housing modifications and the effect of prolonged IRS campaigns over multiple months. Modifications that do not completely remove but simply reduce active ingredient concentrations may still allow for mosquitoes to be killed, but continuous sub-lethal exposures to vectors may lead to other consequences such as the development of resistance [43] and hence the topic warrants further research. Additionally, future studies need to characterize where mosquitoes rest to inform a more targeted surface spraying approach within households [44] and for instance advise home-owners as to which parts of a room may be safe to modify throughout the transmission season and after a spray campaign.

The use of IRS to supplement LLINs for malaria control and elimination is on the rise, and there is sufficient evidence acknowledging the major benefits of IRS and its continued role in malaria control [15, 45, 46]. Our results support other work that demonstrates IRS can be very effective in high transmission settings especially when long-lasting IRS products are deployed [26, 46, 47]. Even though the effective coverage fell to below 60% in Matutuine, in the absence of the IRS campaign, the estimated risk of mosquito bites would have been 2-fold higher. Various studies have been completed [48–50], and more are underway, to expand evidence of the impact of IRS on various disease ecologies and transmission intensities. The ultimate aim of these efforts is to guide program decision-making on when, where, and how to incorporate IRS strategy to maximize benefit [22, 38, 51, 52]. Therefore, assessing any household changes post-IRS application may be an important observation to include in programmatic National Malaria Control Programme (NMCP) monitoring and evaluation to understand where and how we need to close this neglected protective gap in protection. The observations made here are equally important to fairly compare products, as those covering communities that are regularly modifying wall surfaces and/or adding rooms may be spuriously deemed less effective than comparative products in communities making little or no wall modifications and/or adding rooms. Promoting community awareness may in part minimise or shift the timing of modifications, that inadvertently diminish IRS protective effects, and hence greatly improve the expanded benefit of IRS for a community.

## Supporting information

S1 Fig Summary of rooms receiving modification or the addition of rooms to compounds in the November cohort for Matutuine (top panel) and Boane (bottom panel). In each panel, the data are divided by room type into bedrooms, living rooms, kitchens and storage rooms. Shown in grey are the original rooms, noted as the first horizontal bar on each block. The upper limit of the block then shows the total rooms after additions.
(TIFF)

S2 Fig Summary of rooms receiving modification or the addition of rooms to compounds in the December cohort for Matutuine (top panel) and Boane (bottom panel). In each panel, the data are divided by room type into bedrooms, living rooms, kitchens and storage rooms. Shown in grey are the original rooms, noted as the first horizontal bar on each block. The upper limit of the block then shows the total rooms after additions.
(TIFF)

S3 Fig Images of households/rooms where at least one wall is partially covered with fabric. (TIF)

S4 Fig Comparison of factors impacting the IRS effectiveness. Assuming prolonged spray delivery of IRS. **(A-B)**. The relative increase in daily mosquito biting rates in each district assuming prolonged spraying in the absence of housing modification and in the absence of mosquito net use, or presence of mosquito net use, Matutuine (blue) and Boane (green) districts sprayed with an organophosphate and neonicotinoid active, respectively. **(C-D)**. The relative increase in daily mosquito biting rates in each district assuming prolonged spraying in the presence of housing modification and absence of either mosquito net use, or presence of mosquito net use, Matutuine (blue) and Boane (green) districts sprayed with an organophosphate and neonicotinoid active, respectively. 90% uncertainty intervals are carried through the analysis and reflect IRS product efficacy over time, and the uncertainty analysis for parameters shown in Table 2.
(TIF)

S5 Fig Comparison of factors impacting the IRS effectiveness. Assuming overnight spray delivery of IRS. **(A-B)**. The relative increase in daily mosquito biting rates in each district assuming prolonged spraying in the absence of housing modification and in the absence of mosquito net use, or presence of mosquito net use, Matutuine (blue) and Boane (green) districts sprayed with an organophosphate and neonicotinoid active, respectively. **(C-D)**. The relative increase in daily mosquito biting rates in each district assuming prolonged spraying in the presence of housing modification and absence of either mosquito net use, or presence of mosquito net use, Matutuine (blue) and Boane (green) districts sprayed with an organophosphate and neonicotinoid active, respectively. 90% uncertainty intervals are carried through the analysis and reflect IRS product efficacy over time, and the uncertainty analysis for parameters shown in Table 2.
(TIF)

S1 File PLOS’ inclusivity in global research questionnaire. (DOCX)
