# Supplementary figures and images for: Household modifications after the indoor residual spraying (IRS) campaign in Mozambique reduce the actual spray coverage and efficacy
Source: PLOS Glob Public Health. 2022 Apr 20;2(4):e0000227. doi: 10.1371/journal.pgph.0000227 (PMC10021718; doi:10.1371/journal.pgph.0000227)

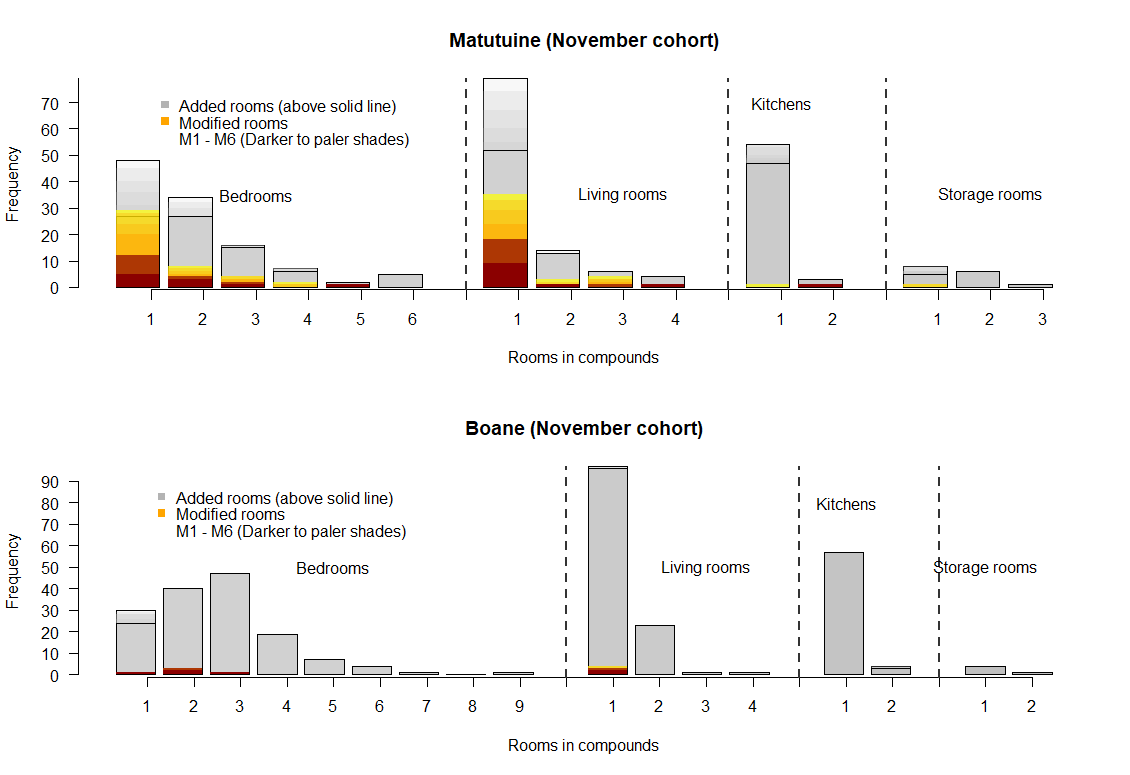

Supplement: S1 Fig — In each panel, the data are divided by room type into bedrooms, living rooms, kitchens and storage rooms. Shown in grey are the original rooms, noted as the first horizontal bar on each block. The upper limit of the block then shows the total rooms after additions. (TIFF) [file pgph.0000227.s001.tiff]

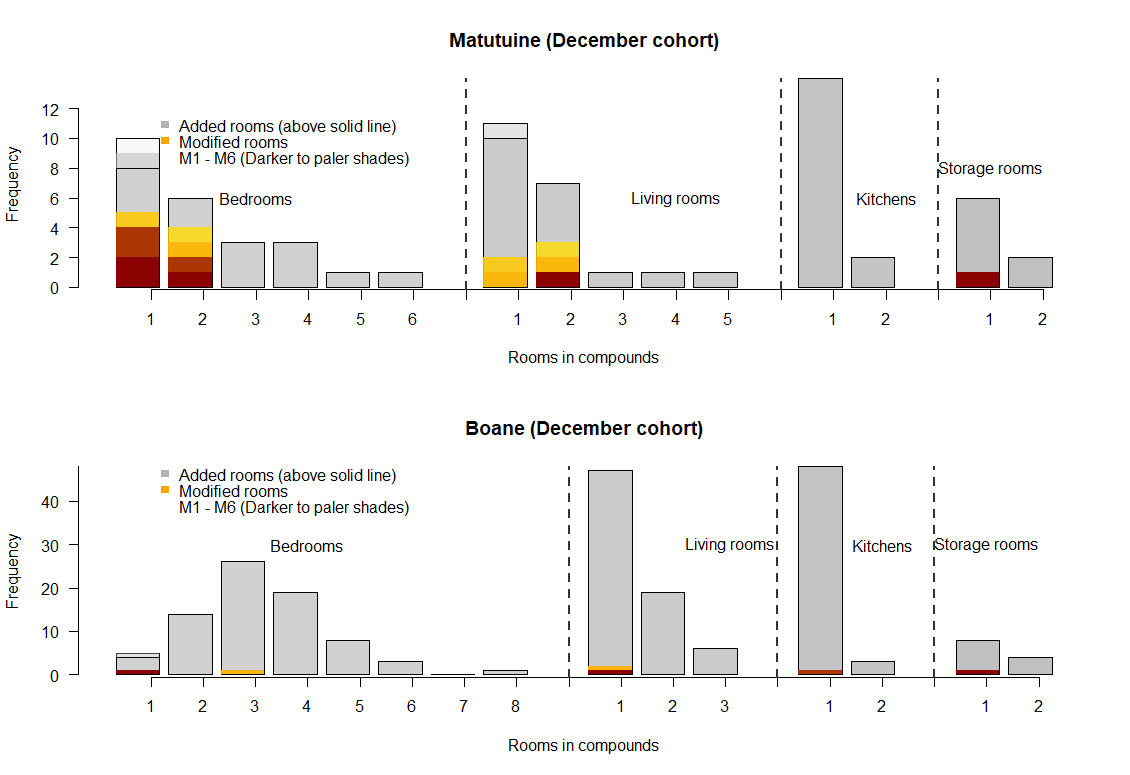

Supplement: S2 Fig — In each panel, the data are divided by room type into bedrooms, living rooms, kitchens and storage rooms. Shown in grey are the original rooms, noted as the first horizontal bar on each block. The upper limit of the block then shows the total rooms after additions. (TIFF) [file pgph.0000227.s002.tiff]

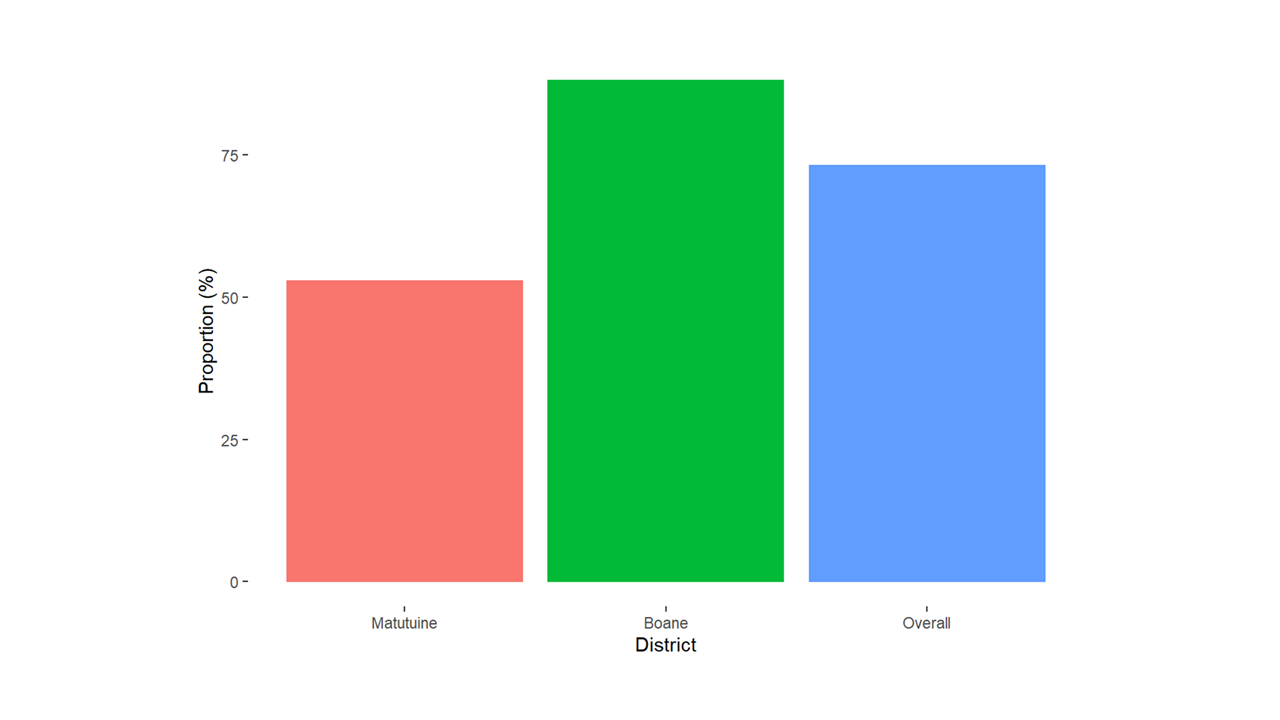

Supplement: S3 Fig — (TIF) [file pgph.0000227.s003.tif]

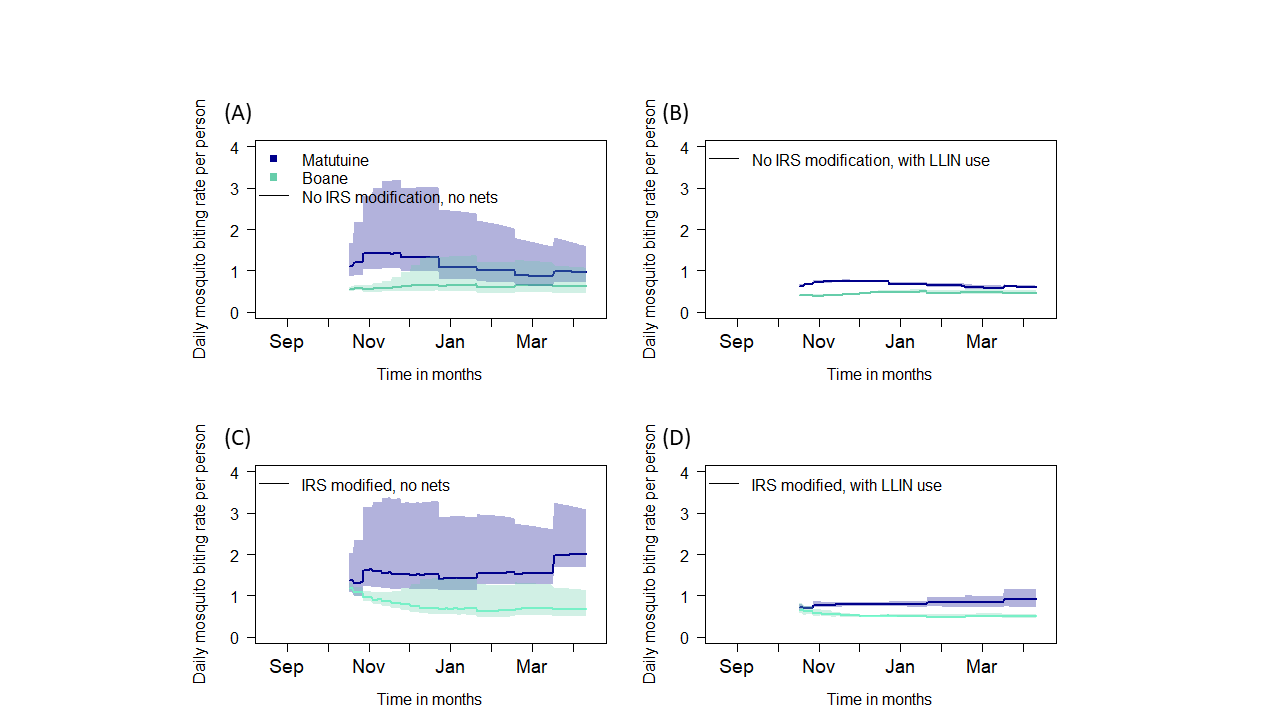

Supplement: S4 Fig — Assuming prolonged spray delivery of IRS. (A-B). The relative increase in daily mosquito biting rates in each district assuming prolonged spraying in the absence of housing modification and in the absence of mosquito net use, or presence of mosquito net use, Matutuine (blue) and Boane (green) districts sprayed with an organophosphate and neonicotinoid active, respectively. (C-D). The relative increase in daily mosquito biting rates in each district assuming prolonged spraying in the presence of housing modification and absence of either mosquito net use, or presence of mosquito net use, Matutuine (blue) and Boane (green) districts sprayed with an organophosphate and neonicotinoid active, respectively. 90% uncertainty intervals are carried through the analysis and reflect IRS product efficacy over time, and the uncertainty analysis for parameters shown in Table 2. (TIF) [file pgph.0000227.s004.tif]

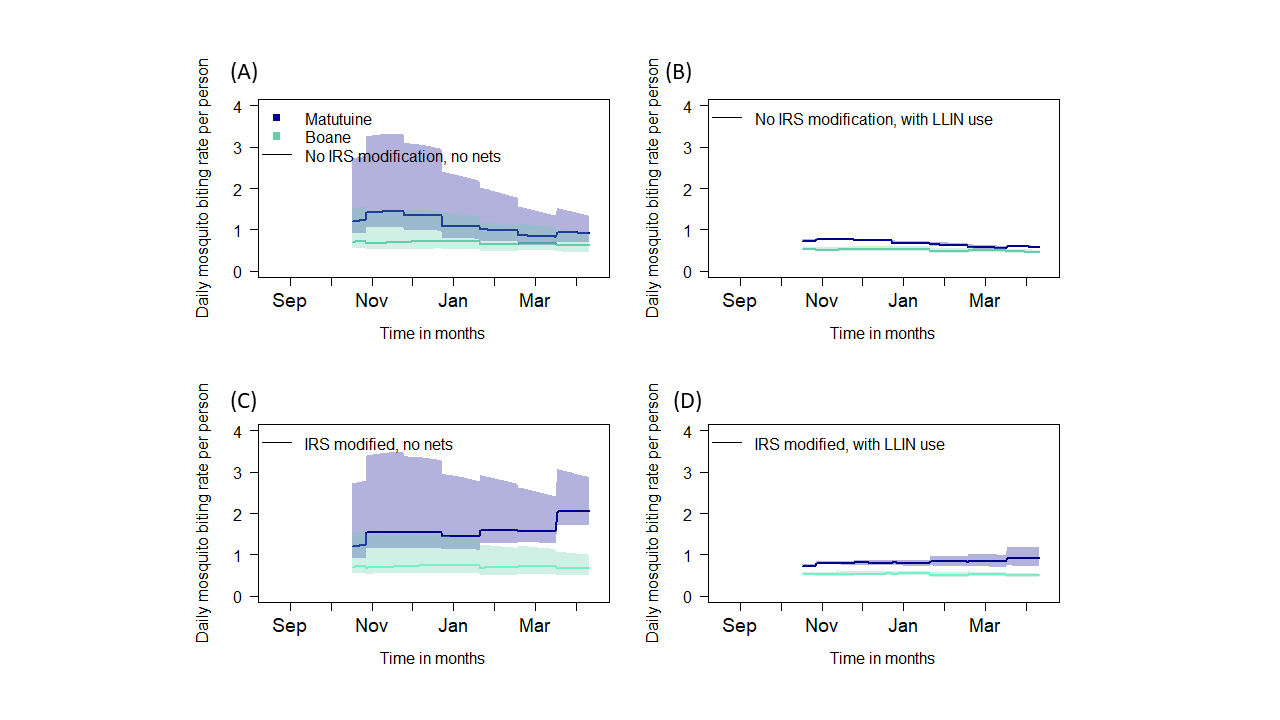

Supplement: S5 Fig — Assuming overnight spray delivery of IRS. (A-B). The relative increase in daily mosquito biting rates in each district assuming prolonged spraying in the absence of housing modification and in the absence of mosquito net use, or presence of mosquito net use, Matutuine (blue) and Boane (green) districts sprayed with an organophosphate and neonicotinoid active, respectively. (C-D). The relative increase in daily mosquito biting rates in each district assuming prolonged spraying in the presence of housing modification and absence of either mosquito net use, or presence of mosquito net use, Matutuine (blue) and Boane (green) districts sprayed with an organophosphate and neonicotinoid active, respectively. 90% uncertainty intervals are carried through the analysis and reflect IRS product efficacy over time, and the uncertainty analysis for parameters shown in Table 2. (TIF) [file pgph.0000227.s005.tif]
